# Supplementary material for: A new targeted capture method using bacterial artificial chromosome (BAC) libraries as baits for sequencing relatively large genes
Source: PLoS One. 2018 Jul 12;13(7):e0200170. doi: 10.1371/journal.pone.0200170 (PMC6042959; doi:10.1371/journal.pone.0200170)
Supplement: S1 Protocol — (DOCX) [file pone.0200170.s002.docx]

**S1 Protocol. Amplification efficiency of two polymerases.**

A 2 μL of 1st post-captured library solution before removing magnetic beads concentrated using a BDC method was used as a template for PCR in a 20 μL solution containing 0.5 U of PrimeSTAR GXL DNA Polymerase (Takara Bio), deoxynucleotide (dNTP) 0.2 mM, 0.2 μM of each primer, Sol_bridge_P5 and Sol_bridge_P7 in Maricic et al. (2010). The PCR and purification were carried out using the same method as BDC with PrimeSTAR. The same volume of the 1st post-captured library solution was used as a template for PCR in a 20 μL solution containing, 0.4 U of KAPA HiFi DNA Polymerase (Kapa Biosystems), deoxynucleotide (dNTP) 0.3 mM, 0.5 μM of each primer, Sol_bridge_P5 and Sol_bridge_P7 in Maricic et al. (2010). PCR was carried out using the following protocol: an initial denaturing step at 98^o^C for 2 min, 16 cycles for the 1st post-capture library of denaturation at 98^o^C for 20 s, annealing at 60^o^C for 30 s, extension at 72^o^C for 45 s, and a final extension step at 72^o^C for 5 min. To determine the technical variability in targeted captures, each PCR was performed in duplicate. The PCR amplicons were quantified using Qubit 3.0 Fluorometer (ThermoFisher Scientific).
